# Perceptions of Uncertainty in Medical Care Among Non-medical Professionals and Nurses in Japan: A Cross-Sectional Internet-Based Preliminary Survey

**DOI:** 10.7759/cureus.55418

**Published:** 2024-03-02

**Authors:** Naomi Akiyama, Shihoko Kajiwara, Ryuji Uozumi, Tomoya Akiyama, Kenshi Hayashida, Jasmine Sim, Mie Morikawa

**Affiliations:** 1 School of Nursing, Nagoya City University, Nagoya, JPN; 2 School of Nursing, Gifu University of Health Sciences, Gifu, JPN; 3 Department of Industrial Engineering and Economics, School of Engineering, Tokyo Institute of Technology, Tokyo, JPN; 4 Center for Postgraduate Clinical Training and Career Development, Nagoya University Hospital, Nagoya, JPN; 5 Department of Medical Informatics and Management, University Hospital of Occupational and Environmental Health, Kitakyushu, JPN; 6 National Institute of Education, Nanyang Technological University, Singapore, SGP; 7 Department of Policy Studies, College of Policy Studies, Tsuda Unversity, Tokyo, JPN

**Keywords:** citizen, nurse, medical care, tolerance, uncertainty

## Abstract

Background: Medical care is impacted by uncertainty caused by various factors. The uncertainty that exists in medical care can cause patient distrust and lead to conflict. This study compared the tolerance of uncertainty in medical care between non-medical professionals and nurses.

Methods: We conducted a cross-sectional Internet-based survey. Participants included 2,100 individuals (600 nurses and 1,500 non-medical professionals; aged ≥ 20 years) from different parts of Japan. Of these, we excluded 70 participants who were classified as non-medical professionals but were registered nurses. Finally, we analyzed data from 2,030 participants (600 nurses and 1,430 non-medical professionals). Three registered nurses and nursing researchers developed an original questionnaire on tolerance of uncertainty in medical care. Data regarding participants’ characteristics (age, sex, education level, marital status, having children, population size of the residential area, medical care usage, and occupation) were obtained. We performed a one-way analysis of variance (ANOVA) to compare the data between non-medical professionals and nurses. Additionally, we employed a multiple regression model to investigate factors related to tolerance of uncertainty in medical care scores.

Results: A significant portion of participants (36.7%) were aged 40-50 years (n = 745). Most were women (n = 1,210, 59.6%), and a considerable percentage were medical care users (n = 1,309, 64.5%). Non-medical professionals were less tolerant of uncertainty than nurses, and uncertainty scores were associated with medical care usage, occupation, and population size of the residential area.

Conclusions: Our findings revealed variations in perceptions of uncertainty in medical care between non-medical professionals and medical care providers. To mitigate conflicts related to medical issues, medical care providers should enhance non-medical professionals' education regarding perceptions of uncertainty in medical care.

## Introduction

Medical accident victims often experience feelings of frustration and anger after such accidents, while medical staff, as secondary victims, may experience fear concerning malpractice, contributing to burnout [1, 2]. Conflicts arising from medical accidents between patients and staff cause frustration for both parties. Medical personnel provide medical care and support to patients, aiming to prevent casualties and medical accidents. However, human factors, including memory lapses and slips, can contribute to errors in practice.

Medical care is impacted by uncertainty caused by various factors. Han et al. clarified that scientific uncertainty encompasses uncertainties about diagnosis, prognosis, causal explanations, and treatment recommendations [3, 4]. Practical uncertainty applies to the structures and processes of care, the competence of one’s physicians, and the rules and procedures of institutions [3, 4]. Mackintosh et al. reported that conceptual uncertainty in healthcare is related to moral and normative dimensions, such as patients’ experiences of diseases or their culture and social practices [5]. Particularly, five factors leading to uncertainty have been identified: patients’ biological variability (e.g., constitutional predisposition), patient and physician bias, error in test interpretation, differing values and opinions of patients and physicians, and uncertainty surrounding decision-making [6]. Divergent perspectives on treatment goals contribute to disagreements between physicians and patients. Patients tend to think that their condition is getting better, which may be because the patient’s wishes are reflected or the patient may not have enough information to set the correct goal. Effective communication of evolving treatment goals by physicians is essential, as changes occur during treatment [7, 8]. Patients often express dissatisfaction with the perceived lack of empathy and insufficient information provided by medical staff, resulting in complaints. Concrete instances include concerns regarding medicines, examinations, and treatment [9].

The World Health Organization (WHO) emphasizes that engaging patients and families in healthcare is critical [10]. However, it is not confirmed whether a gap exists in perceptions and awareness regarding medical care between medical professionals and the public that could lead to complaints and medical litigation. Nurses have the role of advocating for people’s rights, supporting people in making decisions and choices, and spending most of their time interacting with patients. Therefore, the purpose of this study is to compare the tolerance of uncertainty in healthcare between non-medical professionals and nurses. The definition of non-medical professionals in this study excluded people with medical qualifications, such as nurses, public health nurses, physicians, dentists, and pharmacists.

This research paper focuses on the perceptions of non-medical and medical professionals that contribute to patient safety, mutual understanding between patients and medical professionals in clinical settings, and the avoidance of disputes and litigation. The results may help improve communication between patients and healthcare professionals, as well as eliminate misunderstandings in the area of informed consent.

This article was previously posted to the Research Square Preprints server on October 9, 2023, but the preprints were not reviewed and are unofficial.

## Materials and methods

Study design

The study developed a cross-sectional Internet survey for non-medical professionals and nurses aged ≥ 20 years. Members of the survey company who met this study’s eligibility criteria were provided with a document explaining this survey’s purpose, specifically to compare the tolerance of uncertainty in medical care between non-medical professionals and nurses. Those who read the document and expressed interest were included.

Eligibility criteria

Participants were nurses and non-medical professionals aged ≥ 20 years from different parts of Japan. Mandatory criteria were access to a computer or similar devices with an Internet connection and being registered monitors with the Internet survey company.

Study setting

We conducted an Internet-based cross-sectional survey using the platform of an Internet survey company that maintains a database of over three million individuals. The recruitment and survey period lasted two weeks in March 2023.

Ethical considerations

This study was approved by the ethics committee of Gifu University of Health Science, Gifu, Japan (approval no.: 2022_19). We outsourced the Internet survey administration to an online research company, which contacted the survey monitors and collected the study data. The purpose of the survey was provided at the beginning of the questionnaire, and only those who agreed were allowed to proceed with the questions (Q). Informed consent was sought from participants through the Internet system. All methods were performed in accordance with relevant guidelines and regulations. The data were anonymized by the company and subsequently sent to the researchers.

Measures

The survey tool included questions about participant characteristics such as age, sex, education level, marital status, having children, medical care use as inpatient or outpatient at a medical institution, the population size of the residential area, occupation, and their perceptions of uncertainty in healthcare.

Participants were split into three categories based on their age: 20-39 years, 40-59 years, and ≥ 60 years. Education levels were classified as university/graduate school, vocational school/junior college, and junior high school/high school; junior colleges included two- or three-year courses. Marital status was categorized as married, which included divorced and widowed, or unmarried. Child status was categorized as without children and having children, which included those children living with the participant and those not. The population size of the residential area was categorized as follows: capital, including 23 wards of Tokyo or ordinance-designated cities with a population of 500,000 people or more; city, with a population of 200,000 people or more; middle city, with a population of 100,000 people or more; town, with a population of less than 100,000 people; and small town, with a population of less than 10,000 people. Regarding hospitalization or medical institution visit status, binary variables were used; the participant answered “yes” if they were currently an inpatient or outpatient at a medical institution (designated “medical care user”) or “no” if they were not. Occupations were classified as nurses and non-medical professionals. Additionally, occupation details included six categories: nurses, office workers (company workers and civil servants), self-employed (farmers, fishermen, and owners), housewives and students, unemployed, and others.

Questions about perceptions of uncertainty in healthcare were as follows: “Do you think that the saying ‘humans make mistakes’ applies to medical professionals as well?” (Q1); “Do you think that doctors may not be able to make a diagnosis in one visit?” (Q2); “Do you think that there are individual differences in how the effects of treatments and drugs appear?” (Q3); “Do you think that some people will have severe side effects even if they take the same medicine, while others will not?” (Q4); “Do you think that sudden deterioration (sudden worsening of the condition) or death that medical professionals cannot predict will occur?” (Q5); and “What do you think is the probability of neuropathy (numbness, sensation, movement abnormalities, etc.) from blood collection?” (Q6). The responses to Q1 through Q5 were rated on a seven-point Likert scale (absolutely agree: one; absolutely disagree: seven); Q3 and Q5 were reverse-scored. Higher scores indicated a lower tolerance of uncertainty in healthcare, and vice versa.

Item Q6 indicated the probability of neuropathy owing to blood sampling through seven options: one in 100 chances of developing neuropathy; one in 1,000; one in 100,000; one in 1,000,000; one in 10,000,000; one in 100,000,000; and no chance of developing neuropathy.

As shown in Appendix 1, a new questionnaire was created for this study by three nurse researchers based on previous research on uncertainty in healthcare (internal validity). A pre-study was conducted with five people (including registered nurses and non-medical professionals) to test the questionnaire. Based on their feedback, we revised the survey sheet so that it could be more easily understood by respondents. The questionnaire had 25 questions. Participants answered all questions within five to 10 minutes.

Sample size

Participants were randomly sampled from among the monitors of the Internet survey company, stratified according to age, sex groups (in similar proportions), and residential area. Regarding the residential area, the number of participants to be selected from the monitors was determined according to the population composition ratio of the prefecture. The Internet survey company sent a questionnaire to all participants and collected questionnaire responses until the target number was reached. To allow a 6.5% margin of error with a 95% confidence level and assume the population composition published by the Statistics Bureau of the Ministry of Internal Affairs and Communications [11], 228 participants were required for each category constructed by age and sex. The target number of participants was set at 1,430, allowing for invalid responses. For the nurses, the sex ratio of Japanese nurses was biased, with the percentage of female and male nurses being 91.9% and 8.1%, respectively [12]. Therefore, nurses were randomly sampled and stratified according to age. Similarly, 228 participants were calculated for 40-59 years, and fewer participants with a wider margin were set for the remaining age categories owing to a lack of nurses based on a survey of employed nurses by age group in 2020 [12]. The target number of nurses was set at 600 since it was unlikely that medical professionals would give invalid answers.

Analysis

Data were analyzed using t-tests and chi-square tests to compare the scores on the awareness of uncertainty in medical care questionnaires between non-medical professionals and nurses. We estimated the regression coefficients using a multiple regression model. In the model, the medical uncertainty scores of items Q1-Q6 served as the dependent continuous variables, while age, sex, education level, marital status, child status, population size of the residential area, medical care user, and occupations were all independent categorical variables. All statistical analyses were performed using SAS software version 9.4 (SAS Institute, Cary, NC) and Stata version 11.0 (StataCorp LLC, College Station, TX).

## Results

Participants’ flowchart

**Figure 1:**
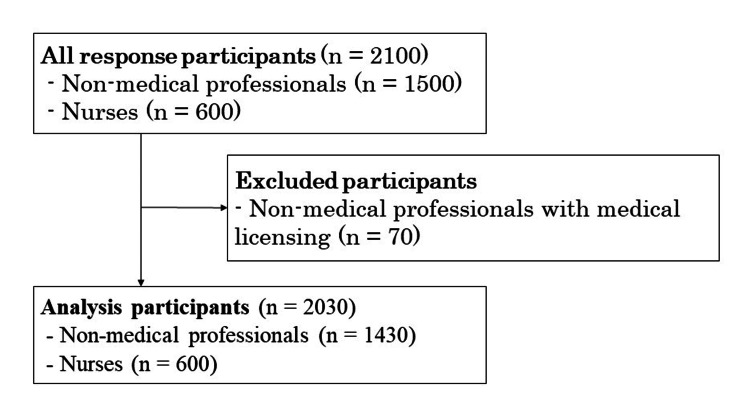
Flowchart showcasing the participant selection process

Data were collected from 2,100 participants, including non-medical professionals (n = 1,500) and nurses who currently work in medical institutions (n = 600). We excluded 70 participants who were classified as non-medical professionals but were medically licensed. Finally, we analyzed data from 2,030 participants (non-medical professionals: 1,430; nurses: 600). Figure 1 presents the participant flowchart.

Study characteristics

**Figure 2:**
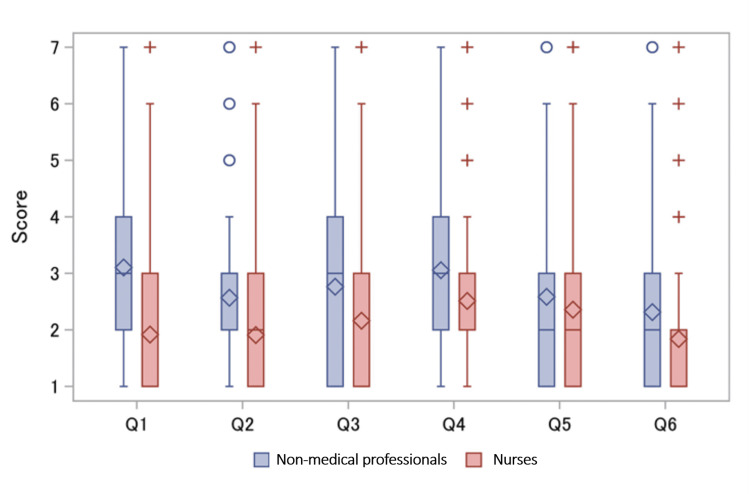
Scores on perceptions of medical uncertainty: comparisons between non-medical professionals and nurses Diamonds represent mean scores. Q1: “Do you think that the saying ‘humans make mistakes’ applies to medical professionals as well?” Q2: “Do you think that doctors may not be able to make a diagnosis in one visit?” Q3: “Do you think that there are individual differences in how the effects of treatments and drugs appear?” Q4: “Do you think that some people will have severe side effects even if they take the same medicine, while others will not?” Q5: “Do you think that sudden deterioration (sudden worsening of the condition) or death that medical professionals cannot predict will occur?” Q6: “What do you think is the probability of neuropathy (numbness, sensation, movement abnormalities, etc.) from blood collection?” Q: question

Table 1 shows the study participants’ characteristics. A higher proportion of non-medical professionals were aged 60 years and older compared to nurses and held a university or graduate school degree. Scores on the perceptions of uncertainty in medical care and comparisons between non-medical professionals and nurses are presented in Figure 2. All the means of non-medical professionals were higher than those of nurses (p <.01 for Q1-Q6).

**Table 1:** Participant characteristics: non-medical professionals and nurses Numbers shown (%); results based on chi-square test.

Particulars	All	Non-medical professionals	Nurses	P-value
		n = 1,430	n = 600	
Age (years)				
20-39	670 (33.0)	470 (32.9)	200 (33.3)	< .001
40-59	745 (36.7)	474 (33.1)	271 (45.2)	
≥ 60	615 (30.3)	486 (34.0)	129 (21.5)	
Sex				
Male	820 (40.4)	725 (50.7)	95 (15.8)	< .001
Female	1210 (59.6)	705 (49.3)	505 (84.2)	
Education level				
University/graduate school	825 (40.6)	664 (46.4)	161 (26.8)	< .001
Vocational school/junior college	726 (35.8)	295 (20.6)	431 (71.8)	
Junior high school/high school	479 (23.6)	471 (33.0)	8 (1.4)	
Marital status				
Unmarried	698 (34.4)	535 (37.4)	163 (27.2)	< .001
Married	1332 (65.6)	895 (62.6)	437 (72.8)	
Child status				
Without children	970 (47.8)	732 (51.2)	238 (39.7)	< .001
Having children	1060 (52.2)	698 (48.8)	362 (60.3)	
Population size in the living area				
Capital	706 (34.8)	509 (35.6)	197 (32.8)	.114
City	472 (23.3)	323 (22.6)	149 (24.8)	
Middle city	332 (16.4)	245 (17.1)	87 (14.5)	
Town	410 (20.2)	272 (19.0)	138 (23.0)	
Small town	110 (5.4)	81 (5.7)	29 (4.8)	
Medical care user				
Yes	721 (35.5)	501 (35.0)	220 (36.7)	.483
No	1309 (64.5)	929 (65.0)	380 (63.3)	
Occupation details				
Nurses	600 (29.6)	-	600 (100.0)	-
Office workers	599 (29.5)	599 (41.9)	-	
Self-employed	72 (3.5)	72 (5.0)	-	
Housewives and students	247 (12.2)	247 (17.3)	-	
Unemployed	254 (12.5)	254 (17.8)	-	
Others	258 (12.7)	258 (18.0)	-	

Table 2 presents the perception of uncertainty scores from the multiple regression model. These present comparisons between the various characteristics of non-medical professionals and nurses. The scores of all the questions were associated with ages 20-39 and occupations. Ages 20-39 and occupation among non-medical professionals were strongly positively associated with Q1-Q6. Small towns (a population size of residential area) were positively associated with all questions except Q3. Moreover, being a medical care user was negatively associated with all questions except Q3. A negative association was noted between male participants and Q1, but junior high school/high school education was positively associated with Q1. The variable without children was negatively associated with the scores of items Q3 and Q5. Appendices 2-3 show scores on perceptions of medical uncertainty between nurses' and non-medical professionals' occupations.

**Table 2:** Multiple regression analysis of the scores on perceptions of medical uncertainty Q: question; coef.: regression coefficient; ref.: reference

	Q1		Q2		Q3		Q4		Q5		Q6	
	Coef.	P	Coef.	P	Coef.	P	Coef.	P	Coef.	P	Coef.	P-value
Age (years) (ref. = 40-59)												
20-39	0.174	0.045	0.209	0.002	0.201	0.015	0.185	0.008	0.272	0.002	0.26	0.004
≥ 60	0.104	0.252	-0.06	0.401	-0.12	0.179	-0.08	0.252	-0.06	0.495	0.187	0.045
Sex (ref. = female)												
Male	-0.33	< .001	-0.11	0.071	-0.07	0.346	0.036	0.568	0.036	0.637	0.142	0.076
Education level (ref. = university/graduate school)												
Vocational school/junior college	0.043	0.631	-0	0.963	0.063	0.459	-0.05	0.486	0.002	0.982	0.004	0.963
Junior high school/high school	0.337	< .001	0.135	0.064	0.008	0.932	0.079	0.172	-0.09	0.343	0.21	0.029
Marital status (ref. = married)												
Unmarried	-0.06	0.585	0.034	0.698	0.093	0.392	0.078	0.387	0.042	0.701	-0.02	0.898
Having children (ref. = having children)												
Without children	-0.16	0.131	-0.1	0.222	-0.26	0.01	-0.11	0.172	-0.3	0.003	0.058	0.590
Population size in the living area (ref. = capital)												
City	-0.03	0.765	0.021	0.774	0.093	0.298	0.008	0.921	0.123	0.186	0.008	0.934
Middle city	0.018	0.866	0.038	0.648	0.158	0.114	0.033	0.699	0.182	0.081	-0.03	0.756
Town	0.137	0.163	0.055	0.473	0.051	0.586	0.048	0.548	0.057	0.559	-0.04	0.726
Small town	0.573	< .001	0.775	< .001	0.215	0.162	0.952	< .001	0.52	0.001	0.057	0.001
Medical care user (ref. = no)												
Yes	-0.19	0.014	-0.24	< .001	-0.13	0.073	-0.29	< .001	-0.2	0.01	-0.31	< .001
Occupations (ref. = nurses)												
Non-medical professionals	1.227	< .001	0.662	< .001	0.314	< .001	0.417	< .001	0.65	< .001	0.393	< .001

## Discussion

This study examined the perceptions of medical uncertainty among non-medical professionals and nurses in Japan. Our study findings are similar to previous results in that a gap exists in the perceptions of patients’ health beliefs between medical care professionals and patients [13]. Healthcare is affected by uncertainty in structures and processes of care and not all situations in medical institutions can be strictly controlled [4]. Street et al. reported that physicians may not necessarily be good judges of patients’ health beliefs and recommended the implementation of partnership-building strategies to encourage active patient participation [13]. Our results revealed that, compared with nurses, non-medical professionals are less tolerant of uncertainty regarding four points: 1) people making mistakes; 2) diagnosis, treatment, or drug uncertainty; 3) sudden deterioration; and 4) injury from blood collection.

To fill the gap between non-medical professionals and healthcare providers, the latter should inform the former regarding any uncertainty in medical care provision, and non-medical professionals should recognize the uncertainties inherent in medical care.

Non-medical professionals are less tolerant of human error

We found that non-medical professionals’ scores on perceptions of medical uncertainty were higher than those of nurses for item Q1, suggesting that non-medical professionals were less accepting of medical uncertainty owing to human errors than nurses. Item Q1 includes the term “humans make mistakes,” stated as a common saying in the fields of health and safety, indicating that human error is natural [14]. The probability of the occurrence of human error cannot be zero; however, we noted a gap of 1.2 points in the scores for item Q1 between non-medical professionals and nurses. In the multiple regression model, after adjusting for other variables, non-medical professionals appear to be less tolerant than nurses of uncertainty in healthcare. Informed consent involves processes through which healthcare providers educate patients about the risks and benefits of and alternatives to treatment procedures or interventions [15]. However, informed consent does not include explanations regarding errors that can occur because healthcare providers are humans. As such, general non-medical professionals may be unaware that human error can occur in healthcare. Therefore, non-medical professionals must be educated that errors cannot be completely prevented, even if healthcare providers confront human errors from an individual or organizational standpoint.

Compared to nurses, non-medical professionals are less tolerant of diagnostic, treatment, or drug uncertainty

Our findings revealed an association between medical care users and their tolerance of medical uncertainty. Moreover, regarding the population size of residential areas, small towns were positively associated with diagnostic uncertainty and side effects of drugs, and the age group of 20-39 years and non-medical professionals were positively associated with diagnostic uncertainty, effects of treatment, and drugs, and side effects of drugs.

Medical litigation includes issues such as intraoperative negligence, inadequate informed consent, ineffective explanations regarding complications, and poor teamwork or communication [16]. Medical providers must communicate with their patients about uncertainties in medical care. Dahm et al. reported that physicians used two main communication strategies to manage diagnostic uncertainty, and patient-centered communication was associated with positive patient reactions [17]. A study of physicians’ tolerance in medical care indicated that tolerance of uncertainty is associated with the characteristics and experience of physicians and that less tolerance is associated with burnout [18], which has been prominently displayed during the coronavirus disease 2019 (COVID-19) pandemic. Although the medical situation in the early days of the COVID-19 pandemic was dire and a standard treatment was lacking, medical care had to be provided. In addition, there was uncertainty about when this situation would end. The ability to tolerate uncertainty was needed to prevent burnout among healthcare providers [19, 20]. Outside of the COVID-19 pandemic, medical care is generally fraught with uncertainty. All treatments and drugs have individual differences in efficiency, and some patients develop serious complications and side effects. To prevent burnout and conflicts related to medical issues, healthcare providers must communicate with patients about uncertainty in medical care.

Prior research has reported an association between tolerance of uncertainty in medical care and the size of residential areas, demonstrating how access to health information and its use differ between rural and urban areas [21]. This also shows that differences in health literacy between rural and urban populations are not atypical and are associated with social factors [22]. Differences based on the population size of residential areas should be examined further in future research.

Non-medical professionals are less tolerant of sudden deterioration

The gap between healthcare providers’ and patients’ perceptions of prognostic uncertainty can be attributed to poor communication between patients and care providers. Examples of poor communication include physicians’ reluctance to communicate the uncertain circumstances that can lead to a patient’s death, while the family prefers to acquire all necessary details regarding the loss of their loved one [23,24]. Akiyama et al. reported that healthcare providers did not have risk perceptions of patients’ accidental choking, although choking has caused severe disabilities or even led to patient deaths [25]. Medical care users demonstrated a tolerance for sudden deterioration, but all occupations, excluding self-employment, and those living in small towns were associated with an intolerance of uncertainty in medical care. We believe that the reasons for this trend are similar to those for the population’s low tolerance of diagnosis, treatment, or drug uncertainty. Healthcare providers cannot fully anticipate all the sudden changes a patient may experience. However, in some cases, preventive efforts can be taken by providing appropriate explanations to patients and their families or by predicting risks and taking necessary measures.

Compared to nurses, non-medical professionals are less tolerant of injuries during blood collection

Nerve injury during venipuncture can be caused by direct puncture or compression [26]. The incidence of nerve injury during venipuncture can vary, with estimates in the range of 0.02%-0.03% [27, 28]. Although the incidence is very low and recovery may occur after a few months, venipuncture is a common medical procedure; hence, public awareness about neuropathy is an important matter to be addressed by healthcare providers. Participants aged 20-39 years, those living in a small town, and those with non-medical occupations demonstrated a lower tolerance of uncertainty in medical care than did other participants. Tolerance of uncertainty in medical care was associated with medical care usage. Younger generations might be perceived as less tolerant because they have less experience using medical care. Non-medical professionals’ tolerance of uncertainty in blood collection is very low in comparison to that of nurses; thus, it is necessary to carefully explain the possibility of neuropathy to non-medical professionals.

Strengths and limitations of the research

Our study had some limitations. First, the characteristics of non-medical professionals and nurses differed. In general, most nurses are women, with research reporting 92.2% female nurses and 8.1% male nurses [29]. Thus, it was impossible to avoid biases related to sex and education level owing to occupational characteristics. Second, we implemented an online survey method. While this approach is convenient for collecting information from many non-medical professionals, information about the population distribution could not be obtained from the Internet survey monitors. In addition, Andrade et al. reported the possibility of respondents having biases toward this type of survey [30]. Thus, our findings may have limited generalization.

However, as a research strength, our study provides a different perspective on non-medical professionals’ tolerance of uncertainty in medical care. Nurses play an important role in medical care as they spend most of their time with patients. Our results demonstrate that perception gaps exist among non-medical professionals and nurses. This gap might be even greater for other medical professionals and non-medical professionals. Collecting data from non-medical professionals using controlled sampling strategies presents some difficulties. Despite these limitations, our study provides a different perspective on non-medical professionals’ tolerance of uncertainty in medical care.

## Conclusions

Our results revealed that non-medical professionals are less tolerant of uncertainty in medical care than nurses are regarding the following four points: there are people making mistakes; diagnosis, treatment, drug uncertainty; sudden deterioration; or injury from blood collection.

The results demonstrate that patients who receive medical care are more tolerant of medical uncertainty than non-patients. In addition, young people were generally less tolerant than older respondents. The younger generation, those who are likely to be family members of patients, have a low tolerance for medical care uncertainty and may even become involved in medical conflicts through their families. Therefore, to reduce conflicts related to medical issues, healthcare providers need to inform non-medical professionals regarding the perception of uncertainty in medical care.

## Figures and Tables

**Figure 3:**
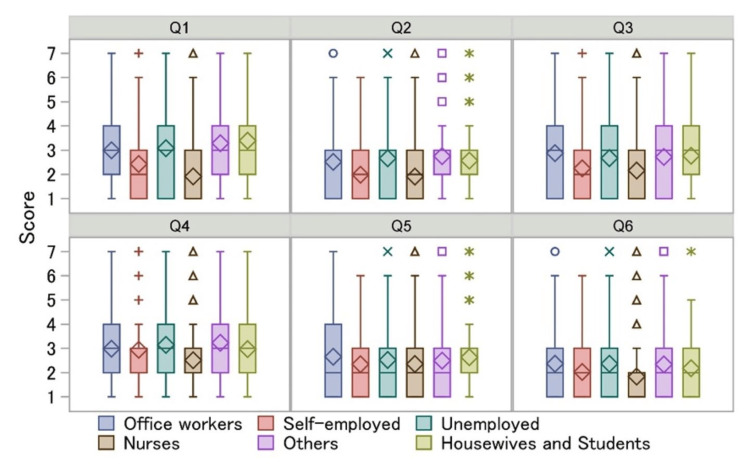
Scores on perceptions of medical uncertainty: comparisons between non-medical professionals' occupation details and nurses Diamonds represent mean scores. Q1: ‘Do you think that the saying “humans make mistakes” applies to medical professionals as well’? Q2: ‘Do you think that doctors may not be able to make a diagnosis in one visit’? Q3: ‘Do you think that there are individual differences in how the effects of treatments and drugs appear’? Q4: ‘Do you think that some people will have severe side effects even if they take the same medicine, while others will not’? Q5: ‘Do you think that sudden deterioration (sudden worsening of the condition) or death that medical professionals cannot predict will occur’? Q6: ‘What do you think is the probability of neuropathy (numbness, sensation, movement abnormalities, etc.) from blood collection’? Q: question

## Appendices

**Table 3:** Questionnaire

Participants were randomly sampled from among the monitors of the Internet survey company and stratified according to age, sex (in similar proportions), and residential area.					
Please choose one appropriate answer to each of the following questions.					
Q1. Please let me know your sex. (single selection)	1. Male				
	2. Female				
Q2. Please let me know your age.	( )-years-old				
Q3. Please let me know your living prefecture. (single selection)	1. Hokkaido	2. Aomori	3. Iwate	4. Miyagi	5. Akita
	6. Yamagata	7. Fukushima	8. Ibaragi	9. Ibaraki	10. Gunma
	11. Saitama	12. Chiba	13. Tokyo	14. Kanagawa	15. Niigata
	16. Toyama	17. Ishikawa	18. Fukui	19. Yamanashi	20. Nagano
	21. Gifu	22. Shizuoka	23. Aichi	24. Mie	25. Shiga
	26. Kyoto	27. Osaka	28. Hyogo	29. Nara	30. Wakayama
	31. Tottori	32. Shimane	33. Okayama	34. Hiroshima	35. Yamaguchi
	36. Tokushima	37. Kagawa	38. Ehime	39. Kochi	40. Fukuoka
	41. Saga	42. Nagasaki	43. Kumamoto	44. Ooita	45. Miyagi
	46. Kagoshima	47. Okinawa			
Please choose one appropriate answer to the following questions.					
Q1. Are you married? (single selection)	1. Unmarried				
	2. Married (separated/widowed)				
Q2. Do you have children? (single selection)	1. Living with children				
	2. We don’t live together, but we have children				
	3. No children				
Q3. Please let me know your occupation. (single selection)	1. Working at a company				
	2. Company management				
	3. Civil servant, teacher, non-profit organization employee				
	4. Self-employed (business and industry services)				
	5. Agriculture, forestry, and fisheries				
	6. Doctor				
	7. Dentist				
	8. Pharmacist				
	9. Nurse				
	10. Assistant nurse				
	11. Public health nurse				
	12. Midwife				
	13. Physical therapist				
	14. Occupational therapist				
	15. Other health-related profession				
	16. Legal management				
	17. Professional such as a lawyer or tax accountant				
	18. Housewife/househusband				
	19. Student				
	20. Other occupation				
Q4. Do you have a medical license? (single selection)	1. Yes				
	2. No				
Q5. Are you currently hospitalized or attending a medical institution (hospital or clinic)? (single selection)	1. Yes				
	2. No				
Q6. Do you have experience working at a medical institution (hospital or clinic)? (single selection)	1. Yes				
	2. No				
Q7. What is the current population size of your area? (single selection)	1. Special wards (23 wards of Tokyo) or ordinance-designated cities with a population of 500,000 or more.				
	2. Core cities or special cities with a population of 200,000 or more				
	3. Cities with a population of 100,000 or more				
	4. Cities with a population of less than 100,000 people				
	5. Towns and villages with a population of less than 10,000 people				
Q8. Please indicate your highest educational background. (single selection)	1. Junior high school graduate				
	2. High school graduate				
	3. Vocational school graduate				
	4. Junior college graduate				
	5. University graduate				
	6. Graduate school				
	7. Others				
Q9. Do you think that the saying “humans make mistakes” applies to medical professionals as well? (single selection)	1. Totally true				
	2. Quite true				
	3. More or less likely				
	4. Neither agree nor disagree				
	5. Rather different				
	6. Quite different				
	7. Completely different				
Q10. Do you think that doctors may not be able to make a diagnosis in one visit? (single selection)	1. Totally true				
	2. Quite true				
	3. More or less likely				
	4. Neither agree nor disagree				
	5. Rather different				
	6. Quite different				
	7. Completely different				
Q11. Do you think that there are individual differences in how the effects of treatments and drugs appear? (single selection)	1. Totally true				
	2. Quite true				
	3. More or less likely				
	4. Neither agree nor disagree				
	5. Rather different				
	6. Quite different				
	7. Completely different				
Q12. Do you think that some people will have severe side effects even if they take the same medicine, while others will not? (single selection)	1. Totally true				
	2. Quite true				
	3. More or less likely				
	4. Neither agree nor disagree				
	5. Rather different				
	6. Quite different				
	7. Completely different				
Q13. Do you think that sudden deterioration (sudden worsening of the condition) or death that medical professionals cannot predict will occur? (single selection)	1. Totally true				
	2. Quite true				
	3. More or less likely				
	4. Neither agree nor disagree				
	5. Rather different				
	6. Quite different				
	7. Completely different				
Q14. What do you think is the probability of neuropathy (numbness, sensation, movement abnormalities, etc.) from blood collection? (single selection)	1.Never (zero)				
	2.1 in 100 people				
	3.1 in 1000 people				
	4.1 to 1 in 100,000 people				
	5. 1 in 1 million people				
	6. 1 in 10 million people				

**Table 4:** Multiple regression analysis of the scores on the perceptions of medical uncertainty segregated by non-medical professionals’ occupations Q: question; coef.: regression coefficient; ref.: reference

		Q1		Q2		Q3		Q4		Q5		Q6	
		Coef.	P	Coef.	P	Coef.	P	Coef.	P	Coef.	P	Coef.	P
Age (years) (ref. = 40-59)													
20-39		0.161	0.064	0.202	0.003	0.189	0.023	0.177	0.012	0.253	0.003	0.267	0.003
≥ 60		0.100	0.287	-0.090	0.198	-0.120	0.190	-0.094	0.214	-0.040	0.633	0.169	0.082
Sex (ref. = female)													
Male		-0.290	0.001	-0.080	0.203	-0.090	0.276	0.007	0.921	0.036	0.671	0.176	0.044
Education level (ref. = university/graduate school)													
Vocational school/junior college		0.037	0.681	-0.010	0.874	0.073	0.395	-0.044	0.544	0.012	0.896	-0.010	0.922
Junior high school/high school		0.316	0.001	0.104	0.159	0.023	0.796	0.084	0.273	-0.070	0.439	0.178	0.068
Marital status (ref. = married)													
Unmarried		-0.040	0.697	0.025	0.771	0.099	0.351	0.068	0.450	0.058	0.600	-0.020	0.083
Having children (ref. = having children)													
Without children		-0.150	0.153	-0.100	0.207	-0.260	0.010	-0.119	0.158	-0.300	0.004	0.056	0.605
Population size in the living area (ref. = capital)													
City		-0.030	0.739	0.021	0.783	0.092	0.302	0.008	0.917	0.120	0.194	0.008	0.934
Middle city		0.013	0.899	0.067	0.744	0.153	0.127	0.026	0.761	0.178	0.087	-0.030	0.759
Town		0.140	0.156	0.053	0.490	0.054	0.565	0.048	0.547	0.062	0.524	-0.040	0.699
Small town		0.571	< .001	0.750	< .001	0.220	0.154	0.943	< .001	0.530	0.001	0.551	0.001
Medical care user (ref. = no)													
Yes		-0.180	0.016	-0.240	< .001	-0.140	0.062	-0.294	< .001	-0.190	0.010	-0.310	< .001
Occupations (ref. = nurses)													
Office workers		1.190	< .001	0.608	< .001	0.378	< .001	0.465	< .001	0.725	< .001	0.301	0.008
Self-employed		0.666	0.001	0.161	0.320	0.165	0.403	0.198	0.237	0.204	0.320	0.287	0.180
Housewives and students		1.308	< .001	0.657	< .001	0.327	0.008	0.349	0.001	0.656	< .001	0.399	0.003
Unemployed		1.215	< .001	0.827	< .001	0.348	0.009	0.531	< .001	0.624	< .001	0.442	0.002
Others		1.308	< .001	0.783	< .001	0.192	0.112	0.402	< .001	0.597	< .001	0.551	< .001
